# Use of Turkey Meat Affected by White Striping Myopathy for the Development of Low-Fat Cooked Sausage Enriched with Chitosan

**DOI:** 10.3390/foods9121866

**Published:** 2020-12-15

**Authors:** Larissa Tátero Carvalho, José M. Lorenzo, Francisco Allan L. de Carvalho, Elisa Rafaela Bonadio Bellucci, Marco Antonio Trindade, Rubén Domínguez

**Affiliations:** 1 Department of Food Engineering, Faculty of Animal Science and Food Engineering (FZEA), University of São Paulo, Pirassununga 13635-900, Brazil; larissa.tcarvalho@hotmail.com (L.T.C.); francisco.allan@usp.br (F.A.L.d.C.); trindadema@usp.br (M.A.T.); 2 Centro Tecnológico de la Carne de Galicia, Rúa Galicia n° 4, Parque Tecnológico de Galicia, San Cibrao das Viñas, 32900 Ourense, Spain; jmlorenzo@ceteca.net; 3 Área de Tecnología de los Alimentos, Facultad de Ciencias de Ourense, Universidad de Vigo, 32004 Ourense, Spain; 4 Department of Food Technology and Engineering, UNESP—São Paulo State University, Street Cristóvão Colombo, 2265, São José do Rio Preto 15054-000, Brazil; elisa.bellucci@unesp.br

**Keywords:** *Pectoralis major*, polysaccharide, meat product, healthy meat, functional food

## Abstract

The main objective of this research was the development of a healthy meat product from turkey meat with white striping myopathy. The effect of adding different proportions of chitosan on the qualitative characteristics, sensory acceptance, and stability of cooked sausages during storage was studied. Three treatments were elaborated (control, 1.5% chitosan, and 3% chitosan), stored for 56 days, and characterized in terms of chemical composition, texture profile analysis, drip and pressure loss analysis, and sensory analysis (after processing; day 0). In the different storage periods (0 and 56 days), the pH value, color, thiobarbituric acid reactive substances (TBARS), and volatile compounds were evaluated. The results showed that the moisture content, lipids, proteins, and weight loss decreased (*p* < 0.05) and the ash content increased (*p* < 0.05) with the addition of chitosan. Similarly, the values of texture parameters (hardness, cohesiveness, gumminess, and chewiness) were higher in the sausages reformulated with chitosan than in control samples. The addition of chitosan increased the pH and yellowness (b*) values and reduced (*p* < 0.05) redness (a*) and lightness (L*) values. The b* values (only in reformulated sausages) and pH increased during storage, while a* showed a significant reduction after 56 storage days. Lipid oxidation (TBARS) was kept below the limits of quantification in all samples and both after processing and 56 storage days. However, when quantifying the lipid-derived volatiles, a clear antioxidant activity of chitosan was observed, which limits the release of these compounds, mainly aldehydes (hexanal and nonanal). Finally, the sensory analysis indicated that, although chitosan treatments received the lowest scores for all attributes, the reformulated samples did not differ from control sausages. Therefore, sausage containing chitosan may represent an interesting alternative for adding value to turkey meats affected by white striping myopathy and, at the same time, develop into a healthy and functional meat product increasing the proportion of fibers in one’s diet.

## 1. Introduction

The food industries worldwide, as a result of the significant demand from consumers, have progressively increased their interest in the development of healthier and safer products [1,2]. Consumers perceive meat products as an important source of dietary fat [3,4]. In addition, this fat is usually an important source of saturated fatty acids and cholesterol [2,5,6]. Therefore the meat industry has been looking for strategies to reduce or modify the lipid profile of said products, with the aim of offering consumers healthy products without compromising their sensory characteristics or stability [2]. Turkey meat is characterized by having an optimal nutritional profile (low fat and cholesterol and favorable fatty acid profile) [7], making consumers consider it a good alternative to other meats. However, precisely due to this high unsaturation of the fat in poultry, they are extremely susceptible to oxidation processes that would lead to a rapid deterioration of this meat [8]. It is also known that fresh turkey meat is prone to rapid spoilage; therefore, food industries are seeking technologies to increase its shelf-life [9]. Moreover, white striping myopathy (WS) is a defect that causes losses in production as a result of decreased sensory, technological, and nutritional quality of breast meat [10]. It is defined by white streaks parallel to the muscle fibers on the ventral surface of the *Pectoralis major* muscles, resulting from structural, morphological, and biochemical changes in muscle tissue [11], classified as moderate or severe degrees, according to the thickness of the streaks. It is an important problem for the poultry industry, requiring control to mitigate unfavorable impacts on processing and consumer acceptability [10]. Therefore, the use of meat with this defect allows the use of meat with low commercial value for the production of a healthy meat product, increasing the competitiveness of the meat industry.

On the other hand, in order to improve their nutritional characteristics and increase the stability of meat products by limiting the use of synthetic additives, several compounds such as phenolic compounds [12,13,14,15,16], carotenoids [17], or dietary fibers [18,19] are among the natural ingredients most commonly added to meat products. In this regard, the meat industry shows a growing interest in chitosan and its possible applications. This compound is a polysaccharide derived from the chitin deacetylation process and composes a large part of the exoskeletons of insects, crustaceans, and fungi cell walls [20,21]. It is non-toxic, biodegradable, and biocompatible [20] and, therefore, approved as “generally recognized as safe” (GRAS) [22]. Moreover, the large amount of by-products and discards produced by the seafood processing industry are a renewable and sustainable source to obtain this compound, helping at the same time to limit the environmental problems that these residues cause [21]. The important antioxidant [23] and antimicrobial capacity (against spoilage and pathogenic bacteria and fungi) [9,20,23,24] make chitosan an ingredient of great interest to the food industry. Additionally, it has an emulsifying and water retention capacity [25] and can improve cooking yield and fat retention [26]. Nutritionally, chitosan has significant functional properties such as anti-inflammatory, immunity-enhancing, antitumor, anticancer ones and reduces the absorption of lipids, and, consequently, it has also a hypocholesterolemic effect [27]. Moreover, it is a natural source of dietary fiber [27], indigestible by intestinal enzymes, which gives it prebiotic effects, stimulating the growth of beneficial bacteria in the gastrointestinal tract [21].

Several authors use chitosan as a potent antioxidant and antimicrobial agent in active films and coatings to increase the shelf-life of meat and meat products [28,29,30,31,32]. Contrarily, the studies using the direct addition of chitosan in the formulation of meat products are very limited [1,22,23,33,34]. Therefore, it is very important to continue studying the effect of the reformulation of meat products with the addition of chitosan, as well as its technological, nutritional, and sensory implications. Moreover, due to the multiple health benefits of this compound, its use in meat products also allows the development of new functional foods.

Based on the above premises, the objective of this study was to evaluate the effects of adding different levels of chitosan to cooked sausage made from turkey meat affected by white striping myopathy (WS) on qualitative characteristics, sensory acceptance, and stability during refrigerated storage, aiming at adding more value to these meats through the development of a healthier meat product.

## 2. Materials and Methods

### 2.1. Cooked Sausages Processing

**Figure 1:**
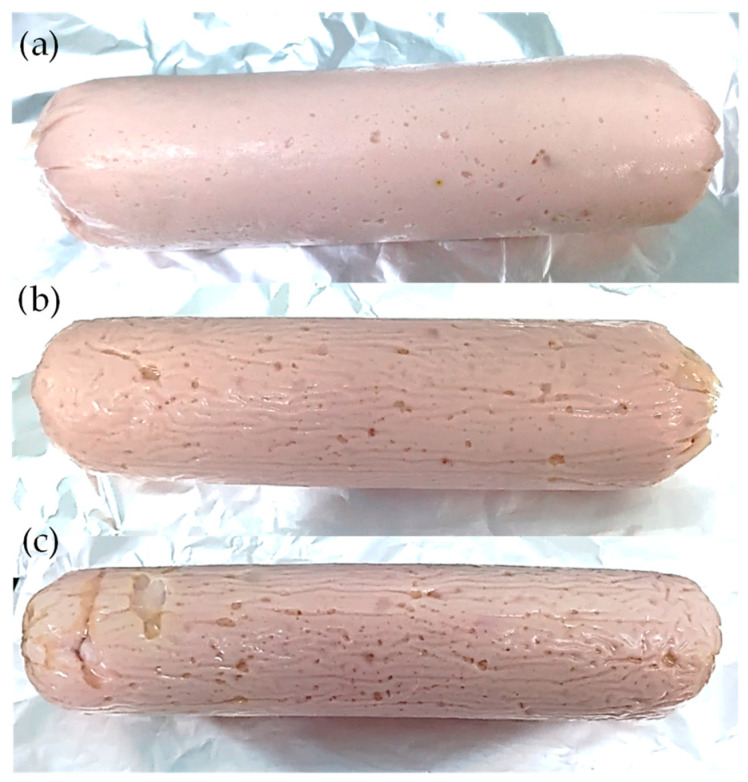
Cooked turkey sausages enriched with chitosan. (Control; (**a**)); 1.5% chitosan (Q1.5%; (**b**)); 3% chitosan (Q3%; (**c**)).

Three batches of cooked sausages were manufactured at the pilot plant of the Centro Tecnológico de la Carne (Ourense, Spain) (Figure 1). A total of 75 sausages, weighing approximately 400 g each (3 treatments × 5 sample points × 5 samples for each sample point) were produced, using turkey meat affected by WS myopathy, with or without chitosan powder (Quimer Vegetable Inputs) with a degree of deacetylation of 91%. The three treatments studied were: (1) control (CONTROL): commercial formulation, without adding chitosan; (2) chitosan 1.5% (Q1.5%): 1.5% chitosan powder in the formulation, and (3) chitosan 3% (Q3%): 3% chitosan powder in the formulation.

The other ingredients used in all formulations were 84.14% turkey breast with WS myopathy in different degrees (50% moderate and 50% severe) acquired in a local slaughterhouse (Ourense, Spain), 1.2% condiment for California ham (Conatril Food Industry, Rio Claro, São Paulo, Brazil), 0.05% sodium erythorbate (Scharlau, Barcelona, Spain), 0.015% sodium nitrite (Scharlau, Barcelona, Spain), 0.25% sodium tripolyphosphate (Scharlau, Barcelona, Spain), and 14.35% water.

For the preparation of sausages, the meat, previously minced in a 6 mm disc, was added with the ingredients, additives, and water, being emulsified to form a homogeneous batter, which was stuffed in 60 mm diameter plastic casings. The meat products were cooked using direct steam until the temperature inside reached 72 °C. The products were immediately chilled in running water and stored at 4 ± 1 °C until the time of analysis.

### 2.2. Proximate Composition Analysis

The procedures described by the International Standards Organization (ISO) were used to determine proteins [35], moisture [36], and ash [37] contents, while the total fat analysis was performed according to the approved procedure Am 05-04 [38].

### 2.3. Color Parameters and pH Analysis

The color was evaluated in a portable colorimeter (CR-600d, Minolta Co. Ltd., Osaka, Japan), using the CIELAB system (L*, a*, and b*) in a device with a pulsed xenon arc lamp, geometry of the 10° viewing angle and 8 mm aperture. The parameters luminosity (L*), red intensity (a*), and yellow intensity (b*) were analyzed.

The pH was obtained using a digital pH meter (Hanna Instruments, Eibar, Spain) with a penetration electrode.

### 2.4. Analysis of Weight Loss by Pressure and Drip Losses

The analysis of weight loss by pressure was determined using 5 g of samples positioned in the middle of two filter papers and between two metal plates. A weight of 2.350 kg was placed on this set for 5 min. Then, the samples were properly weighed to obtain the final weight. The results were calculated as follows:

$$\%\;\mathrm{Weight}\;\mathrm{loss}=\;\frac{\left(\mathrm{initial}\;\mathrm{weight}-\mathrm{final}\;\mathrm{weight}\right)}{\mathrm{initial}\;\mathrm{weight}}\times 100$$

The drip loss analysis was determined using samples cut into 1.5 cm cubes, weighed on a semi-analytical balance (Sartorius/CHE 612) for the initial weight. The samples were placed on a mesh retained on the bottom of a flat plastic container of dimensions approximately 24 × 17 × 7 cm with hermetic closure and kept in a refrigerator (Neveira Liebherr/GKPv 503) at 4 °C for 72 h. Afterward, the samples were properly weighed to obtain the final weight. The results were calculated as follows:

$$\%\;\mathrm{Drip}\;\mathrm{loss}=\;\frac{\left(\mathrm{initial}\;\mathrm{weight}-\mathrm{final}\;\mathrm{weight}\right)}{\mathrm{initial}\;\mathrm{weight}}\times 100$$

### 2.5. Texture Profile Analysis

The texture profile analysis (TPA) was determined by compressing to 60% in sausage cubes (1.0 × 1.0 × 1.0 cm) with a 50 mm cylindrical probe using a Texture Analyzer Texturometer TA-XTPlus. Force–time curves were recorded at a crosshead speed of 3.33 mm/s, following the procedure described by Barros et al. [3].

### 2.6. Lipid Oxidation Determination

Lipid oxidation was evaluated by the TBARS index (substances reactive to thiobarbituric acid; secondary products of lipid oxidation), according to the methodology described by Vyncke [39]. The results were expressed in mg of malondialdehyde/kg of sample.

### 2.7. Volatile Compounds Analysis

The volatile compounds were determined in the cooked sausages along the storage period (0 and 56 days), according to Dominguez et al. [40]. The conditioning, extraction, and injection of the samples were carried out with a PAL-RTC (PalSystem, Schlieren, Switzerland) 120 autosampler. The extractions occurred at a temperature of 37 °C for a period of 30 min, after an initial temperature equilibration at the same temperature, for a period of 15 min. After sampling, to determine the volatiles, the fiber was transferred to the injection port of a gas chromatograph (7890B Agilent Technologies, Santa Clara, CA, USA) equipped with a 5977B mass selective detector (Agilent Technologies, Santa Clara, CA, USA) and a DB-624 capillary column (30 m × 0.25 mm i.d., film thickness 1.4 μm; J&W Scientific, Folsom, CA, USA). The compounds were identified by comparing their mass spectra with those contained in the NIST14 library. The results were defined in units of area (AU) × 10^4^/g of sample.

### 2.8. Sensory Evaluation

The cooked sausages made from turkey meat affected by WS were sensory evaluated on days 0 and 56 of storage. The sessions were conducted by 64 consumers from Ourense, Spain (with ages between 29 and 40 y and from both genders), in the sensory analysis room of the Centro Tecnológico de la Carne, equipped with individual cabin according to regulations [41]. To clean the palate between samples, a cracker without salt and water at room temperature were served. The acceptance test was applied to evaluate the appearance, odor, flavor, texture, and overall quality attributes of each of the samples, using a hedonic scale of 7 points (varying from like very much to dislike very much).

### 2.9. Statistical Analysis

The data were arranged in a completely randomized design with three treatments (CONTROL, Q1.5%, Q3%) and five replications. Fifteen cooked sausages of turkey meat enriched with chitosan were evaluated at each point: five samples of sausages for each batch × three batches (CONTROL, Q1.5%, Q3%). The normal distribution and homogeneity of the variance were previously tested (Shapiro–Wilk). The effects of the treatments were verified using one-way ANOVA, and for the significant effects (*p* < 0.05), the averages were compared by the Tukey test at 5% significance. Statistical analyzes were carried out using SAS (Version 9.4, SAS Institute Inc., Cary, NC, USA).

## 3. Results and Discussion

### 3.1. Initial Composition and Stability during Storage

The proximate composition of cooked turkey meat sausages enriched with chitosan is shown in Table 1. The moisture, lipids, and protein contents in the sausages decreased (*p* < 0.001), while the ash and dry matter contents increased (*p* < 0.001) with the addition of chitosan. This effect was expected, since chitosan was added dry after the initial mixture of the meat mass, possibly causing the diluting effect, reducing the final percentage of moisture, lipids, and proteins but increasing ash and dry matter in the sausages. In contrast to our results, other authors concluded that the compositional parameters did not show significant differences among control and frankfurters with 0.5 and 1% of chitosan [42]. Similarly, in another study, the authors reported that in the initial mass, the proximate composition of fermented cooked sausages did not show differences among control and reformulated sausages with 0.25 and 0.5% of chitosan [1]. However, as occurs in our research, these authors observed that after processing, the sausages containing chitosan presented significantly higher amounts of ash and lower fat than control samples [1]. In a burger model system, the application of different proportions of chitosan (between 0.25 and 1%) did not influence moisture content, while samples with 1% chitosan showed a significant fat content decrease [26]. Thus, taking into account that the portion of chitosan added to the reformulated samples in our study is greater than that used by these authors, it is to be expected, as it happens, that the observed differences will be greater. This fact is confirmed, since in our study, the decrease in fat and moisture and the increase in ash are progressive with the increase in chitosan used in the cooked sausage formulation. Moreover, from a nutritional point of view, the inclusion of chitosan also increases the fiber content of sausages (data not shown; <0.2% in control, 0.70% in Q1.5%, and 1.27% in Q3%). This is an important aspect, since meat products with chitosan dietary fiber are considered functional foods because this fiber is not digestible by intestinal enzymes, which allows them to act as prebiotics [21].

According to the Brazilian Ministry of Health [43], the sausages elaborated in this study can obtain the appeal of “free of fat”, for exhibiting values lower than 0.5 g of fat per 100 g of the product. Similarly, it is also worth mentioning that all formulations can be considered as “high in protein” (at least 20% of the energy value of the food is provided by protein) and “fat-free” (< 0.5 g of fat per 100 g of the product) according to European Regulation [44].

Regarding physicochemical parameters, there was no difference (*p* > 0.05) in the drip losses among treatments (Table 2), with values ranging from 0.80 to 1.57%. On the other hand, the increase in the concentration of chitosan caused a reduction of approximately 75% of the values of weight loss by pressure. These results agree with those reported in previous studies, where the authors observed that the addition of chitosan in emulsified meat-based model food [45,46] and fermented cooked sausages [1] caused a significant reduction in cooking and weight losses. Additionally, the use of 2% of chitosan in fat-reduced meat model also caused a significant reduction in expressible water [46], which agrees with the results of weight loss by pressure obtained in the present research. In the same way, the use of 0.5 or 1% of chitosan in the formulation of burger model system increased cooking yield [26]. It is well known that cooking yield is directly related to water and fat retention in the meat product. These authors have evaluated these retentions and conclude that, like cooking yield, both water and fat retention increase with the inclusion of 0.5 and 1% chitosan in meat batter [26]. The authors argued that this effect was due to the high water holding capacity of chitosan [1,26]. Preliminary researches have indicated that WS myopathy damages the water retention capacity [47,48,49]; therefore, chitosan was able to reduce water loss during storage by avoiding water exudate out of the product. 

According to the texture profile analysis (TPA) described in Table 2, the use of 1.5 and 3% chitosan increased (*p* < 0.05) the value of all parameters (except springiness) compared to the control. It is important to note that the increase in these parameters is progressive, as the chitosan content is increased in the formulation of the sausages. These results agree with those reported by several authors, who observed that the inclusion of chitosan in a cooked meat model system increased hardness [45,46]. Contrarily, other researchers found that the addition of chitosan at 0.2% did not affect the texture of pork sausages [33], while in the fermented cooked sausages, the use of chitosan (0.25 and 0.5%) decreased texture parameters [1]. It should be noted that the studies in which no differences in texture were observed used a much lower proportion of chitosan (< 0.5%) than in the studies in which its use increased the hardness of the meat product (> 1% of chitosan), which could explain the contradictory results obtained by the different authors. This fact was confirmed by a recent study, in which the use of 0.5% in the reformulation of frankfurters did not affect the texture, while the addition of 1% significantly increased the hardness of the sausages [42]. Consistent with our results, several authors have verified that the increase in hardness with the addition of chitosan may be related to the fact that chitosan may act as a binder, thereby favoring the formation of a stronger gel with myosin and entrapping water [42,45].

On the other hand, the addition of chitosan (1.5 and 3%) increased the pH values (*p* < 0.05) compared to the control treatment (Table 3). Likewise, several authors found an equivalent effect of chitosan on the pH of vacuum-packed pork sausages [33] fermented cooked sausages [1], pork model burger [26], or beef patties [34]. This pH increase in the present study and other research is due to the alkaline condition of the chitosan, which agrees with previous authors who reported that the pH of chitosan solutions ranged from 7.2 to 7.8 [33,34].

In contrast to these findings, the use of 0.5 and 1% of chitosan in the reformulation of frankfurters resulted in a pH decrease [42], while the same amounts of chitosan did not influence the pH values in the pork sausages [22]. Additionally, in the present study, there was a significant increment (*p* < 0.05) in pH values during the storage of samples from all treatments. This behavior is consistent with that previously reported by multiple researchers [22,26], while others did not find these increments during the storage of beef patties [34], frankfurters [42], pork sausages [33], or fermented cooked sausages [1]. The increase in pH levels during storage observed in the present study can be attributed to the accumulation of basic compounds derived from proteolytic reactions due to the microbial action [22,26].

Changes in color parameters (L*, a*, and b*) are shown in Table 3. The addition of chitosan to turkey cooked sausages resulted in lower values of L* (*p* < 0.05) and a* (*p* < 0.05) than control. Moreover, at day 0, the reformulation did not affect b* values, while after 56 storage days, the inclusion of chitosan increased these values. Similar results were obtained by other authors in frankfurters, who reported that the chitosan reduced both L* and a*, while a slight increase in b* values was observed [42]. Additionally, in fermented cooked sausages, the inclusion of chitosan decreased a* and increased b* values [1]. These differences in the color parameters between the control and reformulated samples can be attributed to the color (white) of the chitosan [26]. Moreover, the lowest values of L* (a parameter that depends on water distribution) in reformulated cooked sausages could be due to the water binding ability of chitosan [26]. In contrast to these findings, the use of 0.2–1% of chitosan in a pork model burger [26] and 0.2% in pork sausage [33] did not affect a* and b* values.

On the other hand, the storage did not affect L* values in all treatments, while it significantly decreased a* values. It is well known that the redness is related to the meat pigments. Thus, the reduction in red color during storage for all samples can be attributed to the formation of metmyoglobin due to oxidative processes [42]. Regarding the evolution of b* values during storage, control sausages did not suffer any variation, while sausages with chitosan displayed a significantly increase, which agrees with the results obtained by other authors in frankfurters [42] and pork burger model [26]. Different authors suggested that the natural color of the chitosan affected the color of the sausage [33], which increased b* values during the storage.

Lipid oxidation is one of the most important degradative processes in meat products, which produce nutritional and sensory deterioration and, consequently, commercial losses [8]. Several authors found that the addition of chitosan to meat products delay oxidative rancidity during processing and/or storage [22,23,33,34,42,45]. However, other researchers did not find this antioxidative property [1]. The lipid oxidation inhibition properties of chitosan could be due to it acting as a chelator of transition ion metals [42], including free iron that is released from the heme proteins of meat during heat processing [21]. It is well known that these metals have an important role in the initiation stage of lipid oxidation [8], thus the chitosan eliminated the catalyzers in this process. In this study, TBARS values were, in all cases, below the limit of quantification of the analytical method (<0.1 mg malondialdehyde (MDA)/kg), both after processing and during the 56 days of storage. Our results were very similar to those described by Jo et al. [33], who reported that the vacuum-packed samples showed very low and stable oxidation values during storage, not observing differences between the pork sausages reformulated with chitosan and the controls, as well as during the 3-week storage period. Similarly, in a recent study conducted in cooked ham packaged with anaerobic modified atmosphere (70% N_2_/30% CO_2_), the authors also found insignificant oxidation values [50] during 21 days of storage. These authors concluded that both the low fat content of cooked ham and the absence of O_2_ were the two crucial factors for maintaining the lipid oxidation in insignificant values. In fact, it is well known that the fat content (and its composition) is the most important factor in the lipid oxidation process, since lipids are the substrate for the oxidation reactions [8]. Similarly, the O_2_ is a vital component involved in the formation of peroxy radicals (propagation phase) and also a source of reactive oxygen species (ROS) [8]. This completely explains the results obtained in our study, since the sausages were stuffed in plastic (with very low permeability to oxygen) and their formulation contained a very low fat amount (in all cases below 0.32%). Therefore, with all that said, the low fat content and the absence of oxygen determined that lipid oxidation remained at extremely low values (below the limit of quantification).

Volatile compounds derived from lipid oxidation of cooked turkey sausages are shown in Table 4. In both sampling points, after processing and after 56 storage days, five volatiles related to these oxidative reactions were detected. These compounds include one alkane, two aldehydes, and two alcohols, which are considered as markers of secondary oxidation of fatty acids. In this regard, octane, nonanal, and 1-hexanol were derived from oleic acid, 1-butanol from linoleic acid, while hexanal could be derived from oxidation of both, oleic, and linoleic acids [8]. 

The volatile character of these compounds determines their contribution to the appearance of rancid and off-flavor in the meat products [8,40,51]. Among the volatiles detected in this study, hexanal was the greatest indicator of lipid oxidation, since its content increases to a greater extent than that of other compounds [5]. Additionally, it is more stable than other lipid-derived compounds, such as unsaturated aldehydes [8,52]. For several years, multiple authors reported a strong correlation between this aldehyde and other analytical techniques to the measurement of secondary oxidation products or sensory degradation [53,54,55,56]. In fact, some authors use hexanal and other aldehydes instead of other techniques to discuss the oxidation stability of meat products [5,57], since the use of these specific compounds in conjunction with a very sensitive technique (GC-MS) allows a better understanding of oxidative processes than analyzing the samples with other less specific techniques in which various compounds could interfere with the results [8].

In our study, the most important volatile after the processing steps in control samples was hexanal, while in reformulated samples, the octane presented the highest values. After 56 storage days, in all samples, the octane was the most abundant lipid-derived volatile.

Sausages with chitosan inclusion had higher octane values than in control, both after processing and storage. This shows that the release of this compound was more intense in the reformulated samples. Despite this, it should be noted that this compound (together with the rest of the linear alkanes) had little impact on the sensory characteristics of the meat products, since they presented a high detection threshold [40].

On the other hand, the inclusion of chitosan in the cooked sausages had a low influence on the content of alcohols after the processing step. Chitosan slightly increased 1-hexanol amounts, while it did not affect the content of 1-butanol. After 56 storage days, the content of 1-butanol increased in the samples reformulated with 1.5% of chitosan, while the control and 3% chitosan sausages did not show differences between them. In the case of 1-hexanol, reformulated samples showed lower values than control, although this difference was significant only in the sausages formulated with 1.5% of chitosan. These results indicate that there is no specific trend, so the effect that chitosan exerts on the release of alcohols derived from oxidative processes is not clear.

Finally, the inclusion of chitosan limited the release of aldehydes, which have very low odor thresholds and contribute greatly to the final flavor of the meat product [40]. As discussed before, aldehydes are better indicators of lipid oxidation than other volatile compounds. In our case, the chitosan effect against the release of hexanal was dose-dependent, since the concentration of this compound decreased as the chitosan content increased. This effect was observed in both sampling points (after processing and storage). This fact is very important, as hexanal has grassy and fresh notes at low levels and a strong rancid smell at high concentrations [40]. So being able to control its release allows limiting the appearance of undesirable flavors. Similarly, the content of nonanal was lower in the samples formulated with chitosan than in control sausages. After processing, the control samples had twice the content of nonanal than the chitosan samples. This difference was even greater after 56 storage days, since in the control samples, the content of nonanal remained stable, while in the reformulated samples, its content decreased dramatically during storage. This decrease during storage, as occurs in the hexanal content in the sausages of all treatments, is due to the secondary oxidation products degraded to other compounds [8]. This fact agrees with those reported by other authors, who concluded that the decrease in some aldehydes (heptanal an octanal) during dry-fermented sausage storage was due to further oxidative changes [32]. In the same way as our results, these authors also found that the application of a chitosan–oregano coating to traditional dry-fermented sausages reduced the contents of several aldehydes (propanal, pentanal, and hexanal) [32], which demonstrate the protective effect of this coating. Additionally, this fact resulted in better odor and flavor characteristics of sausages after 7 months of storage [32]. Other authors observed that the amount of hexanal was approximately two-fold lower in the meat-based food matrix containing chitosan than in control samples [45]. This fact fully agrees with our results and confirms the antioxidant power of chitosan, as has been observed by numerous authors in previous studies [22,23,33,34,42,45].

### 3.2. Sensory Evaluation

**Figure 2:**
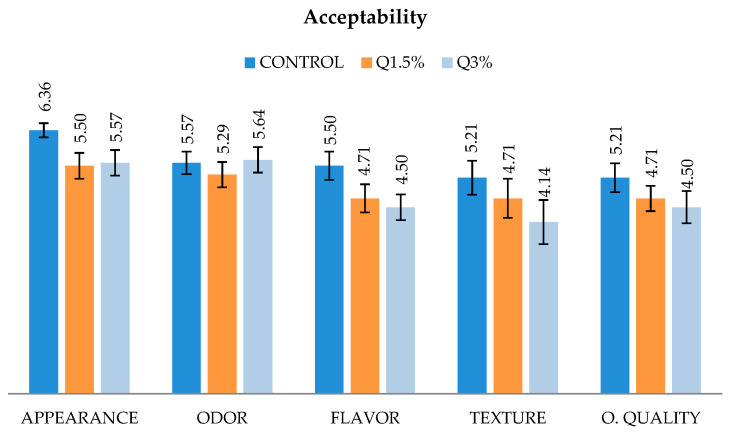
Sensory scores attributed on day 0 by the panel participants for appearance, odor, flavor, texture, and overall quality (O. quality) of low-fat cooked sausages made from turkey meat affected by white striping myopathy enriched with chitosan. Hedonic scale used: 1 = dislike very much; 2 = dislike moderately; 3 = dislike slightly; 4 = neither like nor dislike; 5 = like slightly; 6 = like moderately; 7 = like very much.

From the sensory analysis performed on the cooked turkey sausage with WS myopathy enriched with chitosan (Figure 2), there were no differences (*p* > 0.05) in acceptability parameters, and all treatments received a score above 4, indicating that the addition of chitosan did not alter the acceptability of sausages.

Our results agree with those reported by other authors, who found that the addition of 0.2% of chitosan to pork sausages did not influence any sensory parameters [33]. Additionally, the presence of chitosan in turkey samples did not negatively influence the taste [9]. In fact, these authors concluded that chitosan dipping of turkey filets produced a very pleasant taste, favoring the freshness and natural aroma of the product [9].

In other research, the use of chitosan in the reformulation of pork sausages provides a more satisfactory odor and taste than control samples [22,58]. Similarly, the inclusion of 1% of chitosan in frankfurters showed an increase in color, texture, and overall acceptability scores [42], and the use of 0.5–1% of chitosan in burger model system improved the visual appearance of this product [26]. Additionally, in minced meat, the use of 2% of chitosan was comparable to the application of potassium sorbate and resulted in the remarkable enhancement of meat sensory characteristics [24]. Contrary to these findings, the use of 0.25 or 0.5% of chitosan in the reformulation of fermented cooked sausages negatively impacted the color, aroma, flavor, texture, and overall acceptance scores [1].

In general, taking into account our results and the published studies on the reformulation of meat products with chitosan, it can be stated that its application does not affect or improve the sensory characteristics of final products. However, it is very important to take into account both the amount of chitosan used as well as the meat product to which it was added.

## 4. Conclusions

The addition of chitosan to cooked turkey meat sausages affected by the white striping myopathy caused changes in some of the evaluated technological and quality parameters. Reformulated sausages presented better nutritional characteristics (lower fat content) and oxidative stability (lower aldehydes) than control samples. Additionally, chitosan improved both emulsion stability (lower weight losses) and texture parameters. In contrast, a lower redness value was observed in the reformulated sausages, but any of these differences influenced the sensory characteristics of the cooked sausages. Therefore, as a general conclusion, low-fat sausages containing chitosan may represent an interesting alternative for adding value to turkey meats affected by white striping myopathy and, at the same time, develop into a healthy and functional meat product, increasing the proportion of fibers in one’s diet. The use of chitosan in the sausage formulation has great potential for the enhancement of stability and preservation of this product. However, further studies are necessary to evaluate the influence of chitosan inclusion on the microbial stability of cooked turkey sausages as well as its addition in other meat products. 

## Figures and Tables

**Table 1:** Effect of the chitosan inclusion on chemical composition of low-fat cooked sausages made from turkey meat affected by white striping myopathy.

Proximate Composition (g/100g)	Treatments			SE	Sig.
	CONTROL	Q1.5%	Q3%		
Protein	21.25 ^a^	20.69 ^b^	20.27 ^b^	0.13	***
Lipids	0.31 ^a^	0.14 ^b^	0.08 ^b^	0.03	***
Moisture	76.20 ^a^	75.58 ^ab^	74.98 ^b^	0.16	**
Ashes	2.14 ^c^	2.88 ^b^	3.39 ^a^	0.14	***

^a–c^ The mean values on the same line with different letters indicate a significant difference by the Tukey test; SE: standard error; Sig.: significance; ** *p* < 0.01; *** *p* < 0.001.

**Table 2:** Effect of the chitosan inclusion on drip loss, water loss by pressure, and texture profile of low-fat cooked sausages made from turkey meat affected by white striping myopathy.

Parameters	Treatments			SE	Sig.
	CONTROL	Q1.5%	Q3%		
Drip loss (%)	0.80	1.57	1.01	0.14	ns
Weight loss (%)	15.99 ^a^	4.01 ^b^	3.77 ^b^	1.54	***
Hardness (N)	22.74 ^c^	27.93 ^b^	32.44 ^a^	1.21	***
Cohesiveness	0.56 ^b^	0.67 ^a^	0.67 ^a^	0.01	***
Springiness (mm)	0.86	0.86	0.86	0.00	ns
Gumminess (N)	12.74 ^c^	18.82 ^b^	21.76 ^a^	1.11	***
Chewiness (N.mm)	10.88 ^c^	15.97 ^b^	18.72 ^a^	0.91	***

^a–c^ The mean values on the same line with different letters indicate a significant difference by the Tukey test. SE: standard error; Sig.: significance; ns: not significant; *** *p* < 0.001.

**Table 3:** Effect of the chitosan inclusion on lipid oxidation (TBARS), pH, and color parameters of low-fat cooked sausages made from turkey meat affected by white striping myopathy.

Parameter	Treatments	Storage Days		SE	Sig.
		0	56		
TBARS	CONTROL	-	-	-	-
	Q1.5%	-	-	-	-
	Q3%	-	-	-	-
	SE	-	-	-	-
	Sig.	-	-	-	-
pH	CONTROL	5.99 ^bB^	6.11 ^bA^	0.03	*
	Q1.5%	6.73 ^aB^	6.95 ^aA^	0.04	***
	Q3%	6.77 ^aB^	6.99 ^aA^	0.04	***
	SE	0.10	0.11	-	-
	Sig.	***	***	-	-
Lightness (L*)	CONTROL	80.53 ^a^	80.84 ^a^	0.09	ns
	Q1.5%	74.82 ^b^	74.75 ^b^	0.07	ns
	Q3%	74.41 ^b^	74.14 ^c^	0.11	ns
	SE	0.75	0.81	-	-
	Sig.	***	***	-	-
Redness (a*)	CONTROL	4.13 ^aA^	3.94 ^aB^	0.05	*
	Q1.5%	3.72 ^aA^	3.10 ^bB^	0.12	***
	Q3%	3.23 ^bA^	2.48 ^cB^	0.16	**
	SE	0.12	0.17	-	-
	Sig.	***	***	-	-
Yellowness (b*)	CONTROL	10.19	10.16 ^c^	0.04	ns
	Q1.5%	10.53 ^B^	11.60 ^bA^	0.22	**
	Q3%	10.76 ^B^	12.30 ^aA^	0.29	***
	SE	0.13	0.25	-	-
	Sig.	ns	***	-	-

^A,B^ The average values on the same line with different capital letters indicate a significant difference by the Tukey test; ^a–c^ the mean values in the same column with different lowercase letters indicate a significant difference by the Tukey test; SE: standard error; Sig.: Significance; ns: not significant; * *p* < 0.05; ** *p* < 0.01; *** *p* < 0.001; (-): thiobarbituric acid reactive substances (TBARS) values < 0.1 mg malondialdehyde (MDA)/kg.

**Table 4:** Effect of the chitosan inclusion on lipid-derived volatile compounds (expressed as units of area (AU) × 10^4^/g of a sample) of low-fat cooked sausages made from turkey meat affected by white striping myopathy.

Volatile Compound	Days	Treatments			SE	Sig.
		CONTROL	Q1.5%	Q3%		
Octane	0	12.63 ^bB^	23.02 ^bA^	20.12 ^bAB^	1.43	***
	56	43.52 ^a^	53.77 ^a^	55.63 ^a^	4.61	ns
	Sig.	***	***	***		
Nonanal	0	17.36 ^A^	9.85 ^aB^	9.55 ^aB^	0.93	***
	56	17.34 ^A^	1.27 ^bB^	3.75 ^bB^	0.85	***
	Sig.	ns	***	***		
Hexanal	0	25.42 ^aA^	12.28 ^aAB^	9.10 ^aB^	1.24	***
	56	4.33 ^bA^	3.60 ^bA^	2.11 ^bB^	0.37	**
	Sig.	***	***	***		
1-Butanol	0	5.14 ^b^	4.15 ^b^	4.80 ^b^	0.52	ns
	56	9.96 ^aB^	27.95 ^aA^	9.27 ^aB^	2.11	***
	Sig.	**	***	**		
1-Hexanol	0	1.56 ^bB^	2.03 ^bB^	2.83 ^bA^	0.25	**
	56	19.06 ^aA^	10.70 ^aB^	15.63 ^aA^	1.54	**
	Sig.	***	***	***		

^A,B^ The average values on the same line with different capital letters indicate a significant difference by the Tukey test; ^a,b^ The mean values in the same column with different lowercase letters indicate a significant difference by the Tukey test; SE: standard error; Sig.: Significance; ns: not significant; ** *p* < 0.01; *** *p* < 0.001.

## References

[1] Ozaki M.M., Munekata P.E.S., Lopes A.d.S., Nascimento M.d.S.d., Pateiro M., Lorenzo J.M., Pollonio M.A.R. (2020). Using chitosan and radish powder to improve stability of fermented cooked sausages. Meat Sci..

[2] Domínguez R., Munekata P.E., Pateiro M., López-Fernández O., Lorenzo J.M. (2021). Immobilization of oils using hydrogels as strategy to replace animal fats and improve the healthiness of meat products. Curr. Opin. Food Sci..

[3] Barros J.C., Munekata P.E.S., de Carvalho F.A.L., Pateiro M., Barba F.J., Domínguez R., Trindade M.A., Lorenzo J.M. (2020). Use of tiger nut (*Cyperus esculentus* L.) oil emulsion as animal fat replacement in beef burgers. Foods.

[4] Vargas-Ramella M., Munekata P.E.S., Pateiro M., Franco D., Campagnol P.C.B., Tomasevic I., Domínguez R., Lorenzo J.M. (2020). Physicochemical Composition and Nutritional Properties of Deer Burger Enhanced with Healthier Oils. Foods.

[5] Vargas-Ramella M., Munekata P.E.S., Gagaoua M., Franco D., Campagnol P.C.B., Pateiro M., Barretto A.C.d.S., Domínguez R., Lorenzo J.M. (2020). Inclusion of Healthy Oils for Improving the Nutritional Characteristics of Dry-Fermented Deer Sausage. Foods.

[6] Vargas-Ramella M., Pateiro M., Barba F.J., Franco D., Campagnol P.C.B., Munekata P.E.S., Tomasevic I., Domínguez R., Lorenzo J.M. (2020). Microencapsulation of healthier oils to enhance the physicochemical and nutritional properties of deer pâté. LWT.

[7] Gálvez F., Domínguez R., Pateiro M., Carballo J., Tomasevic I., Lorenzo J.M. (2018). Effect of gender on breast and thigh turkey meat quality. Br. Poult. Sci..

[8] Domínguez R., Pateiro M., Gagaoua M., Barba F.J., Zhang W., Lorenzo J.M. (2019). A comprehensive review on lipid oxidation in meat and meat products. Antioxidants.

[9] Vasilatos G.C., Savvaidis I.N. (2013). Chitosan or rosemary oil treatments, singly or combined to increase turkey meat shelf-life. Int. J. Food Microbiol..

[10] Kuttappan V.A., Brewer V.B., Apple J.K., Waldroup P.W., Owens C.M. (2012). Influence of growth rate on the occurrence of white striping in broiler breast fillets. Poult. Sci..

[11] Kuttappan V.A., Shivaprasad H.L., Shaw D.P., Valentine B.A., Hargis B.M., Clark F.D., McKee S.R., Owens C.M. (2013). Pathological changes associated with white striping in broiler breast muscles. Poult. Sci..

[12] Lorenzo J.M., Pateiro M., Domínguez R., Barba F.J., Putnik P., Kovačević D.B., Shpigelman A., Granato D., Franco D. (2018). Berries extracts as natural antioxidants in meat products: A review. Food Res. Int..

[13] Munekata P.E.S., Domínguez R., Franco D., Bermúdez R., Trindade M.A., Lorenzo J.M. (2017). Effect of natural antioxidants in Spanish salchichón elaborated with encapsulated n-3 long chain fatty acids in konjac glucomannan matrix. Meat Sci..

[14] Lorenzo J.M., Vargas F.C., Strozzi I., Pateiro M., Furtado M.M., Sant’Ana A.S., Rocchetti G., Barba F.J., Dominguez R., Lucini L. (2018). Influence of pitanga leaf extracts on lipid and protein oxidation of pork burger during shelf-life. Food Res. Int..

[15] Pateiro M., Vargas F.C., Chincha A.A.I.A., Sant’Ana A.S., Strozzi I., Rocchetti G., Barba F.J., Domínguez R., Lucini L., do Amaral Sobral P.J. (2018). Guarana seed extracts as a useful strategy to extend the shelf life of pork patties: UHPLC-ESI/QTOF phenolic profile and impact on microbial inactivation, lipid and protein oxidation and antioxidant capacity. Food Res. Int..

[16] Munekata P.E.S., Rocchetti G., Pateiro M., Lucini L., Domínguez R., Lorenzo J.M. (2020). Addition of plant extracts to meat and meat products to extend shelf-life and health-promoting attributes: An overview. Curr. Opin. Food Sci..

[17] Domínguez R., Gullón P., Pateiro M., Munekata P.E.S., Zhang W., Lorenzo J.M. (2020). Tomato as potential source of natural additives for meat industry. A review. Antioxidants.

[18] Bis-Souza C.V., Pateiro M., Domínguez R., Lorenzo J.M., Penna A.L.B., da Silva Barretto A.C. (2019). Volatile profile of fermented sausages with commercial probiotic strains and fructooligosaccharides. J. Food Sci. Technol..

[19] Bis-Souza C.V., Pateiro M., Domínguez R., Penna A.L.B., Lorenzo J.M., Silva Barretto A.C. (2020). Impact of fructooligosaccharides and probiotic strains on the quality parameters of low-fat Spanish *Salchichón*. Meat Sci..

[20] Hu Z., Gänzle M.G. (2019). Challenges and opportunities related to the use of chitosan as a food preservative. J. Appl. Microbiol..

[21] Hamed I., Özogul F., Regenstein J.M. (2016). Industrial applications of crustacean by-products (chitin, chitosan, and chitooligosaccharides): A review. Trends Food Sci. Technol..

[22] Soultos N., Tzikas Z., Abrahim A., Georgantelis D., Ambrosiadis I. (2008). Chitosan effects on quality properties of Greek style fresh pork sausages. Meat Sci..

[23] Kanatt S.R., Chander R., Sharma A. (2008). Chitosan and mint mixture: A new preservative for meat and meat products. Food Chem..

[24] Tayel A.A., Ibrahim S.I.A., Al-Saman M.A., Moussa S.H. (2014). Production of fungal chitosan from date wastes and its application as a biopreservative for minced meat. Int. J. Biol. Macromol..

[25] Lee S.H. (1996). Effect of chitosan on emulsifying capacity of egg yolk. J. Korean Soc. Food Nutr..

[26] Sayas-Barberá E., Quesada J., Sánchez-Zapata E., Viuda-Martos M., Fernández-López F., Pérez-Alvarez J.A., Sendra E. (2011). Effect of the molecular weight and concentration of chitosan in pork model burgers. Meat Sci..

[27] Xia W., Liu P., Zhang J., Chen J. (2011). Biological activities of chitosan and chitooligosaccharides. Food Hydrocoll..

[28] Cardoso G.P., Dutra M.P., Fontes P.R., Ramos A.d.L.S., Gomide L.A.d.M., Ramos E.M. (2016). Selection of a chitosan gelatin-based edible coating for color preservation of beef in retail display. Meat Sci..

[29] Arslan B., Soyer A. (2018). Effects of chitosan as a surface fungus inhibitor on microbiological, physicochemical, oxidative and sensory characteristics of dry fermented sausages. Meat Sci..

[30] Qin Y.Y., Yang J.Y., Lu H.B., Wang S.S., Yang J.Y., Yang X.C., Chai M., Li L., Cao J.X. (2013). Effect of chitosan film incorporated with tea polyphenol on quality and shelf life of pork meat patties. Int. J. Biol. Macromol..

[31] Siripatrawan U., Noipha S. (2012). Active film from chitosan incorporating green tea extract for shelf life extension of pork sausages. Food Hydrocoll..

[32] Krkić N., Šojić B., Lazić V., Petrović L., Mandić A., Sedej I., Tomović V. (2013). Lipid oxidative changes in chitosan-oregano coated traditional dry fermented sausage Petrovská klobása. Meat Sci..

[33] Jo C., Lee J.W., Lee K.H., Byun M.W. (2001). Quality properties of pork sausage prepared with water-soluble chitosan oligomer. Meat Sci..

[34] Suman S.P., Mancini R.A., Joseph P., Ramanathan R., Konda M.K.R., Dady G., Yin S. (2010). Packaging-specific influence of chitosan on color stability and lipid oxidation in refrigerated ground beef. Meat Sci..

[35] ISO 937 (1978). International Standards Meat and Meat Products—Determination of Nitrogen Content.

[36] ISO 1442 (1997). International Standards Meat and Meat Products—Determination of Moisture Content.

[37] ISO 936 (1998). International Standards Meat and Meat Products—Determination of Ash Content.

[38] AOCS (2005). AOCS Official Procedure Am5-04. Rapid Determination of Oil/Fat Utilizing High Temperature Solvent Extraction.

[39] Vyncke W. (1975). Evaluation of the direct thiobarbituric acid extraction method for determining oxidative rancidity in mackerel. Fette Seifen Anstrichm..

[40] Domínguez R., Purriños L., Pérez-Santaescolástica C., Pateiro M., Barba F.J., Tomasevic I., Campagnol P.C.B., Lorenzo J.M. (2019). Characterization of Volatile Compounds of Dry-Cured Meat Products Using HS-SPME-GC/MS Technique. Food Anal. Methods.

[41] UNE-EN ISO 8589:2010/Amd 1:2017 (2017). Sensory Analysis—Methodology—Ranking.

[42] Alirezalu K., Hesari J., Nemati Z., Munekata P.E.S., Barba F.J., Lorenzo J.M. (2019). Combined effect of natural antioxidants and antimicrobial compounds during refrigerated storage of nitrite-free frankfurter-type sausage. Food Res. Int..

[43] Brazil Resolução RDC n° 27, de 13 de janeiro de 1998 (1998). Regulamento Técnico referente à Informação Nutricional Complementar (declarações relacionadas ao conteúdo de nutrientes). Diário Oficial da União; Poder Executivo, de 13 de janeiro de 1998.

[44] (2006). EC Regulation (EC) No 1924/2006 of the European Parliament and of the Council of 20 December 2006 on nutrition and health claims made on foods. Off. J. Eur. Union.

[45] Han M., Clausen M.P., Christensen M., Vossen E., Van Hecke T., Bertram H.C. (2018). Enhancing the health potential of processed meat: The effect of chitosan or carboxymethyl cellulose enrichment on inherent microstructure, water mobility and oxidation in a meat-based food matrix. Food Funct..

[46] Han M., Bertram H.C. (2017). Designing healthier comminuted meat products: Effect of dietary fibers on water distribution and texture of a fat-reduced meat model system. Meat Sci..

[47] Petracci M., Mudalal S., Bonfiglio A., Cavani C. (2013). Occurrence of white striping under commercial conditions and its impact on breast meat quality in broiler chickens. Poult. Sci..

[48] Mudalal S., Babini E., Cavani C., Petracci M. (2014). Quantity and functionality of protein fractions in chicken breast fillets affected by white striping. Poult. Sci..

[49] Mudalal S., Lorenzi M., Soglia F., Cavani C., Petracci M. (2015). Implications of white striping and wooden breast abnormalities on quality traits of raw and marinated chicken meat. Animal.

[50] Pateiro M., Domínguez R., Bermúdez R., Munekata P.E.S., Zhang W., Gagaoua M., Lorenzo J.M. (2019). Antioxidant active packaging systems to extend the shelf life of sliced cooked ham. Curr. Res. Food Sci..

[51] Lorenzo J.M., Carballo J. (2015). Changes in physico-chemical properties and volatile compounds throughout the manufacturing process of dry-cured foal loin. Meat Sci..

[52] Barriuso B., Astiasarán I., Ansorena D. (2013). A review of analytical methods measuring lipid oxidation status in foods: A challenging task. Eur. Food Res. Technol..

[53] Domínguez R., Pateiro M., Agregán R., Lorenzo J.M. (2017). Effect of the partial replacement of pork backfat by microencapsulated fish oil or mixed fish and olive oil on the quality of frankfurter type sausage. J. Food Sci. Technol..

[54] Domínguez R., Gómez M., Fonseca S., Lorenzo J.M. (2014). Influence of thermal treatment on formation of volatile compounds, cooking loss and lipid oxidation in foal meat. LWT Food Sci. Technol..

[55] Domínguez R., Gómez M., Fonseca S., Lorenzo J. (2014). Effect of different cooking methods on lipid oxidation and formation of volatile compounds in foal meat. Meat Sci..

[56] Rivas-Cañedo A., Nuñez M., Fernández-García E. (2009). Volatile compounds in Spanish dry-fermented sausage “salchichón” subjected to high pressure processing. Effect of the packaging material. Meat Sci..

[57] Domínguez R., Pateiro M., Munekata P.E.S., Campagnol P.C.B., Lorenzo J.M. (2017). Influence of partial pork backfat replacement by fish oil on nutritional and technological properties of liver pâté. Eur. J. Lipid Sci. Technol..

[58] Roller S., Sagoo S., Board R., O’Mahony T., Caplice E., Fitzgerald G., Fogden M., Owen M., Fletcher H. (2002). Novel combinations of chitosan, carnocin and sulphite for the preservation of chilled pork sausages. Meat Sci..

